# Biodiversity patterns of epipelagic copepods in the South Pacific Ocean: Strengths and limitations of current data bases

**DOI:** 10.1371/journal.pone.0306440

**Published:** 2024-07-11

**Authors:** Manuela Pérez-Aragón, Ruben Escribano, Reinaldo Rivera, Pamela Hidalgo

**Affiliations:** 1 Doctoral Program of Oceanography, Facultad de Ciencias Naturales y Oceanográficas, Universidad de Concepción, Concepción, Chile; 2 Instituto Milenio de Oceanografía (IMO), Universidad de Concepción, Concepción, Chile; 3 Department of Oceanography, Facultad de Ciencias Naturales y Oceanográficas, Universidad de Concepción, Concepción, Chile; University of Connecticut, UNITED STATES OF AMERICA

## Abstract

Basin-scale patterns of biodiversity for zooplankton in the ocean may provide valuable insights for understanding the impact of climate change and global warming on the marine ecosystem. However, studies on this topic remain scarce or unavailable in vast regions of the world ocean, particularly in large regions where the amount and quality of available data are limited. In this study, we used a 27-year (1993–2019) database on species occurrence of planktonic copepods in the South Pacific, along with associated oceanographic variables, to examine their spatial patterns of biodiversity in the upper 200 m of the ocean. The aim of this study was to identify ecological regions and the environmental predictors explaining such patterns. It was found that hot and cold spots of diversity, and distinctive species assemblages were linked to major ocean currents and large regions over the basin, with increasing species richness over the subtropical areas on the East and West sides of the South Pacific. While applying the spatial models, we showed that the best environmental predictors for diversity and species composition were temperature, salinity, chlorophyll-a concentration, oxygen concentration, and the residual autocorrelation. Nonetheless, the observed spatial patterns and derived environmental effects were found to be strongly influenced by sampling coverage over space and time, revealing a highly under-sampled basin. Our findings provide an assessment of copepods diversity patterns and their potential drivers for the South Pacific Ocean, but they also stress the need for strengthening the data bases of planktonic organisms, as they can act as suitable indicators of ecosystem response to climate change at basin scale.

## Introduction

Spatial patterns of biodiversity in the marine environment have long been documented [1, 2]; however, factors and processes controlling such patterns remain unclear [3]. In the framework of conservation purposes and preserving ocean biodiversity, it becomes clear that the variation in the biogeographic regions from time series observations [4], as well as from geological time scale analyses [5], requires a better understanding of underlying mechanisms driving the spatial and temporal biodiversity patterns [6].

Zooplankton are a significant component of the marine ecosystem; they are the most widespread form of animal life on Earth, with the longest history of evolutionary continuity [7]. These organisms are also the foundation of life in the ocean, acting as the trophic link between primary producers and upper trophic levels [8], being important components of marine biogeochemical cycles [9]. They are also known to rapidly respond to oceanographic and environmental variations, such as fluctuations on temperature [10], oxygenation [11], acidification [12, 13], stratification [14, 15], primary production [16, 17], upwelling [10, 18, 19], circulation [17] and advection [20], thus providing themselves essential environmental sensors for a changing ocean.

Copepods are the taxon with the largest amount of accepted species described and documented in the World Register of Marine Species (WoRMS) [21]. It is also a well-represented taxonomic group in several data bases for marine species, such that a relatively thorough data-base on their distribution (horizontal and vertical) is available for the South Pacific Ocean [22]. Most copepod data bases only report species occurrences, or at higher taxonomic levels, and with emphasis on Calanoid copepods. Even that, such data can facilitate studies on the drivers controlling copepods species richness, how they are spatially structured, and the way they might respond to a changing environment. However, the study of large-scale copepod diversity patterns and their fluctuations driven by environmental forces have been scarcely conducted [23–25]. Moreover, open ocean studies on biogeography patterns of copepods are especially scarce in the Southern Hemisphere. For the vast South Pacific Ocean (SPO), that represents the largest yet the least-known marine ecosystem in the world, available data on copepods are mostly compiled in the Ocean Biodiversity Information System (OBIS) Portal [26]. An important task, however, to use these data sets for studies on copepods distribution and diversity, is the assessment of their quality in terms of resolution and coverage, both in time and space.

With respect to global distribution of plankton species richness, efforts have been made to test hypotheses explaining spatial patterns [23, 27]. For instance, the species-energy hypothesis [28, 29], stating that available energy can regulate species diversity, has been widely supported [30], but how diversity becomes linked to available energy is matter of debate [23]. In the same context, the best predictor of plankton diversity in the ocean appears to be temperature, exhibiting the greatest correlation or highest explanatory power as reported in many works [3, 23, 25, 30–35], although, when analyzing latitudinal patterns of copepod diversity in the Atlantic Ocean, Woodd-Walker et al. (2002) [36] found that species diversity was fundamentally related to variability in seasonality from the equator to polar regions rather than to temperature. The explanation was that more stable conditions and continuous primary production at low latitude can promote a higher diversity, while a highly variable and strongly seasonal cycle of primary production in high latitudes can favor the dominance of fewer species. In any case, the positive correlation between sea temperature and diversity of zooplankton continues to be a key issue for predicting large-scale spatial variability of plankton communities [37, 38]. Temperature is one of the two most important properties of seawater controlling its density and thus governing the dynamics and circulation of the water masses [39]. Temperature also plays a key role in the range of distribution of various marine organisms [34, 40]. For instance, copepods exhibit a strong dependence in their vital rates with temperature [17], since they are ectotherms, so that their population dynamics is strongly influenced by this factor, with major implications on their species composition and diversity patterns [18, 41].

Over a large-scale domain (e.g., a basin-scale), the role of temperature or other environmental correlates as biogeographic predictors for copepods is still unclear for the South Pacific region. The remaining questions are: do copepod species exhibit significant clustering or distinctive spatial assemblies? and, what are the most plausible environmental correlates to predict the diversity distribution of copepods at a basin scale? Lastly, is temperature alone the best predictor of such spatial structures, and whether such structures and biogeographic patterns replicate those observed in the North Pacific or Atlantic Ocean? In this study, we addressed these questions using a long-term (1983–2019) data base on occurrence of copepod species, extracted from the OBIS Portal, allowing us to further evaluate the robustness of these compiled data on species records of epipelagic copepods. For this, we assessed the diversity patterns of planktonic Copepoda and their relationship with oceanographic conditions in the upper 200 m of the western and eastern sides of the SPO to identify biogeographic regions and the presence of hot and cold spots of diversity. We aimed at testing the hypothesis that water temperature is the best predictor explaining spatial diversity patterns of planktonic copepods at basin scale in the SPO. Ultimately, our study will provide a first assessment of copepods diversity for the entire basin, helping us to gain insights into how such patterns may vary under ongoing ocean warming and, also allowing us to evaluate the robustness of current data bases for plankton organisms in this large ocean basin.

## Materials and methods

### Study area

**Fig 1:**
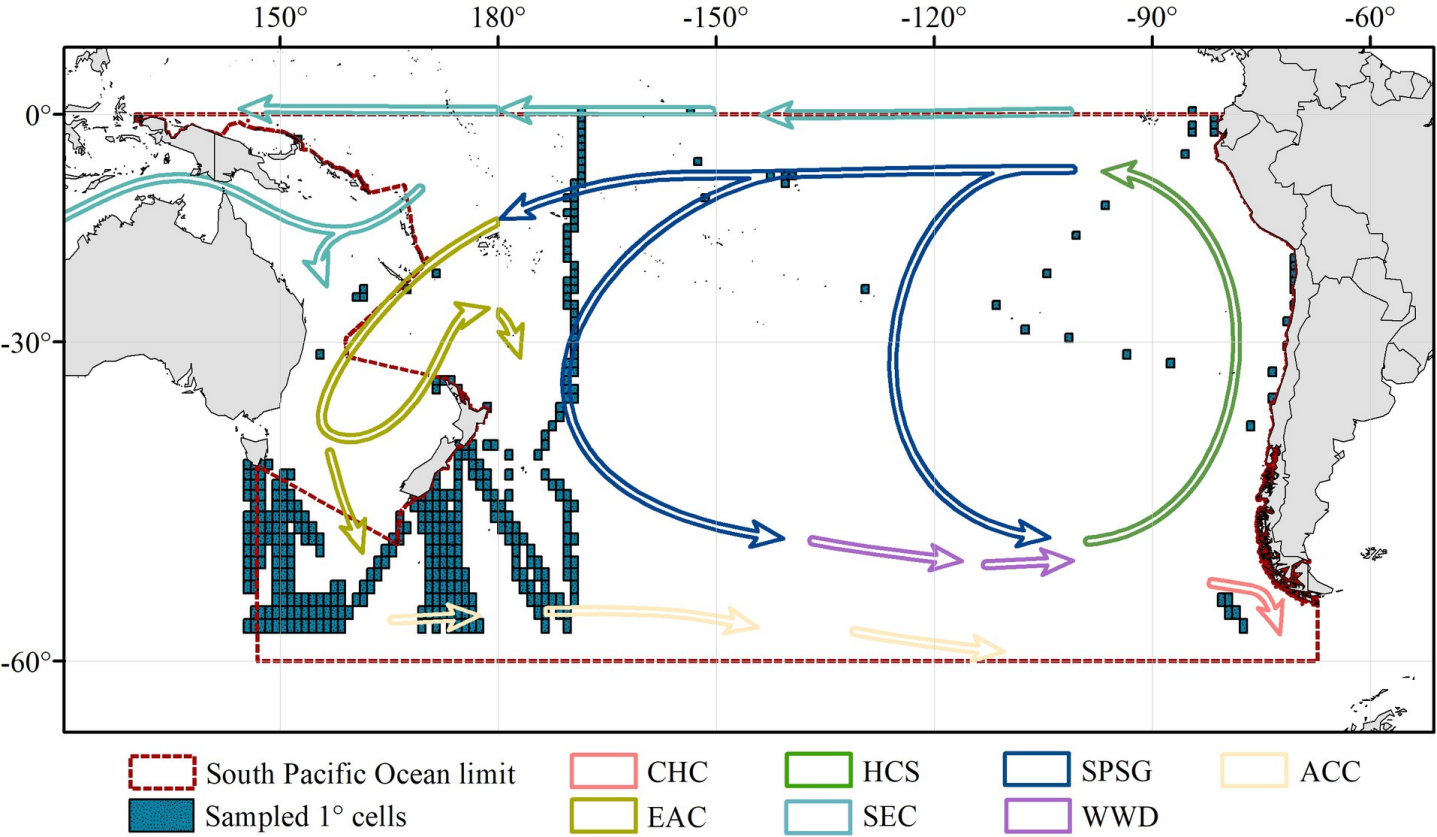
The South Pacific Ocean basin (red dashed area), its sampling coverage with georeferenced locations of the 393 cells (1° x 1°) which were generated (blue squares) from data spanning between 1993–2019. The major near-surface currents shown for the whole basin were obtained from Harvard Dataverse (https://doi.org/10.7910/dvn/tkgo2z). CHC = Cape Horn Current; EAC = East Australian Current; HCS = Humboldt Current System; SEC = South Equatorial Current; SPSG = South Pacific Subtropical Gyre; WWD = West Wind Drift; ACC = Antarctic Circumpolar Current. Map projection is WGS 84/PDC Mercator (EPSG 3832).

The SPO possesses the greatest surface and seabed area, as well as the greatest volume, with a mean depth of 3993 m [42]. The ocean stretches from the Equator—including the islands of the Gilbert and Galápagos Groups, which lie to the northward thereof—until the parallel of 60°S, and between South America and the northeastern limit of the East Indian Archipelago (from New Guinea to the Equator), along the southern, eastern and northern limits of the Bismarck and Solomon Seas; the southeastern and northeastern limits of the Coral Sea; and the southern, eastern and northern limits of the Tasman Sea, going down the meridian of 146°55’E starting at the South East Cape, the southern point of Tasmania, up to the parallel of 60°S [43, 44] (Fig 1). The general circulation in the SPO and major current systems are illustrated in Fig 1.

### Data sources—Copepoda species and quality control procedures

Species occurrence records were obtained from the Ocean Biodiversity Information System, OBIS [22] (http://www.iobis.org) data base, using the robis [45] and devtools [46] packages implemented in the R Core Team (2021). The data were obtained for all occurrences between 0–200 m of the SPO, from 1993 to 2019. Following the retrieval of the data, we eliminated records without information on the geographic coordinates, coordinates equal to zero, or records located inside continents. We selected only records at level of species and excluded duplicate records. This procedure allowed us to compile data to estimate species diversity, but also resulted in a drastic reduction of information since many records are reported for genera or higher taxonomic levels (e.g. families), and in some cases without clear or wrong georeference. Taxonomy was revised and updated using the WoRMS portal (http://www.marinespecies.org) through the *match_taxa* function of robis package [45] implemented in R software (R Core Team, 2021). After we had cleaned and curated the data, a total of 22345 records of Copepoda were selected. The data on occurrences of Copepoda species are available in a Zenodo repository: https://doi.org/10.5281/zenodo.8257222 (version 1). Finally, diversity indexes (see below) were generated based on biogeographic information of copepod biodiversity patterns, implemented in QGIS 3.10 (QGIS, 2022) at 1 degree resolution, totaling 393 cells (Fig 1). However, for all the spatial analyses, the basin was divided into two sections where more data were available. i.e., eastern and western side, excluding a longitudinal band containing cells between -110° and -170°, that had low data coverage (only 16 cells available for that area) and could skew spatial patterns results. Therefore, 377 cells were considered for the analyses. Moreover, when representing alpha diversity patterns through kriging and an inverse distance weighted technique, all sampling cells containing less than 10 species were excluded, assuming they underestimated the species records and hence introduced biases in the patterns. Therefore, for those analyses, only 77 cells were considered.

### Data sources—Environmental database and processing

The oceanographic variables used for analyses were obtained from Copernicus Marine Environment Monitoring Service (https://marine.copernicus.eu) to a resolution of 1 x 1 degree and 0.25 x 0.25 degrees. From this data source, we obtained sea surface temperature [47], salinity [47], total chlorophyll-a concentration [48], dissolved oxygen concentration [48], and mixed layer depth [49]. The resolution selected to analyze our data was 1 degree; hence, the environmental variables with a resolution of 0.25 degrees were resampled into a 1-degree resolution using QGIS 3.10 (QGIS, 2022). All data were averaged for the 0–200 m depth range, as well as from years 1993 to 2019. For temperature data, two additional variables were generated: temperature stability and standard deviation of temperature, which were calculated based on temperature data in temporal lags of two years, with the package climateStability [50] implemented in R software (R Core Team, 2021). “Deviation” of temperature is defined as the mean standard deviation between time slices over time elapsed, which was calculated at the beginning and end of each of the two years temporal lags and dividing this result by the length of the interval to quantify deviation over time for each time slice, to finally average the result across all time slices. Later, “stability” was obtained when taking the inverse of the deviation and scaling it between 0 and 1 [50]. Therefore, a total of seven variables were included in our analyses and are available in Zenodo repository: https://doi.org/10.5281/zenodo.8257222 (version 1). These environmental variables were preprocessed by standardizing them subtracting their mean and dividing them by their standard deviation for later performing variable selection by using Random Forests through the package VSURF [51] implemented in R software (R Core Team, 2021). This is a two-stage strategy based on a preliminary ranking of the explanatory variables using the random forests permutation-based score of importance [52]. We selected a subset of important variables from the second or the “interpretation” step according to the reduction of the out-of-bag error by adding them into the decision training models. These variables were later used for analysis of predictive models of species richness and species composition. Further, for the selected environmental variables, we calculated the Spearman rank-order correlation coefficient matrix to visualize their degree of association as well as check for redundancy.

### Spatial analyses

To analyze the species composition and its spatial distribution, the most dominant species (most recurrent) were extracted from the data bases and then associated with major current systems of the SPO, assuming they were representing distinctive species assemblages from these currents. Also, for beta diversity and its components, turnover and nestedness, a presence-absence matrix (PAM) was generated through the package fuzzySim in R software (R Core Team, 2021) [53] from the list of species that occurred for each range of values. This PAM was later matrix plotted in PAST software [54], from which we selected visually the dominant species, i.e., the ones occurring in all ranges of values for each component.

Biodiversity hotspots were determined based on spatial-statistical criteria to describe biodiversity patterns for the 0–200 m depth water column. It was specifically aimed to detect cells or groups of cells (i.e., spatial clusters) with species richness greater than the richness expected in a random distribution. First, the evaluation of whether copepods depict spatial autocorrelation using Moran’s I statistic was conducted. Second, spatial hotspots were defined in ArcMap 10.4.1 (ESRI 2016) software using the High/Low Clustering (Getis-Ord G*) statistic [55], which identifies spatial concentrations of an entity (in this case species richness per cell) or areas that contain higher/lower values than expected by chance for a given study area. For a hot spot, the observed G* index indicated high values of richness clustered in the study area; whereas for a cold spot, the observed G* index indicated that low values of richness were clustered in the study area.

Species richness (alpha diversity) is the absolute number of species living in a given area, giving equal weight to all resident species [56]. Species composition (namely beta diversity) can be viewed as a measure that compares inventory diversity at two different scales (alpha and gamma diversity). Beta diversity is also a measure of similarity between sites [57], and thus reflects the differences between local biological communities within a region. These differences arise from two phenomena which should be distinguished when understanding how biological diversity is distributed: nestedness and spatial turnover [58]. Nestedness of species occurs when the biota of sites with smaller numbers of species are subsets of the biota at richer sites. Contrary to nestedness, spatial turnover implies the replacement of some species by others as a consequence of environmental sorting or historical and spatial constraints [59]. An application of knowing these components of beta diversity is the attempt to use differences between communities to determine biogeographical regions, as a community that is a subset of a larger one (nestedness) has no exclusive species, and thus such difference should not be taken into account to delimit regions; whereas by using indexes not affected by nestedness it is possible to delimit regions with unique biological communities [58]. We therefore calculated alpha diversity as species richness and beta diversity as species composition while distinguishing between turnover and nestedness. Alpha diversity was calculated in Biodiverse 3.1 software [60], whereas beta diversity was estimated using the packages betapart [61], CommEcol [62] and letsR [63] in R software (R Core Team, 2021), and using the following equation:

$$\beta sor=\beta sim+\beta sne\equiv \frac{b+c}{2a+b+c}=\frac{b}{b+a}+\left(\frac{c-b}{2a+b+c}\right)\left(\frac{a}{b+a}\right),$$ (1)

where βsor is Sørensen dissimilarity, βsim is Simpson dissimilarity (i.e., turnover component of Sørensen dissimilarity), βsne is the nestedness component of Sørensen dissimilarity, a is the number of shared species between two cells, b the number of species unique to the poorest site, and c the number of species unique to the richest site.

As biodiversity index, we used the Shannon-Wiener index which was calculated in Biodiverse 3.1 software [62], according to Eq 2 [64] in the following way:

$$H=-\sum_{i=1}^{n}p_{i}∙ln\;p_{i}$$ (2)

where *p*_*i*_ is the number of samples (in this case, occurrences) of the *i* species as a proportion of the total number of occurrences in the neighborhoods (1° x 1° sampling cells). This proportion is estimated as:

$$p_{i}=\frac{n_{i}}{N}$$ (3)

where *n*_i_ is the number of records of the *i* species and *N* is the total number of records at species level in the sampled cell.

In order to account for the low sampling coverage and explore whether cells with low values of richness were causing biases in the analyses, we discarded the cells with values of alpha diversity < 10, and later performed the spatial analysis using an inverse distance weighted (IDW) technique that interpolates a raster surface from points, limiting to the range of the values used to interpolate and giving more (or less) weight where there is more (or less) data. In addition, to explore further at higher taxonomic levels, we performed the same analysis considering genus and family levels. Both analyses were plotted through Kriging interpolation using ArcMap 10.4.1 software (ESRI 2016).

Since species richness in a sample is highly dependent on sample size or sampling efforts [65], and the ocean has under-sampled areas due to spatial biases in the distribution of sampling locations [6], a skew on the apparent patterns of marine species richness can be produced. Hence, redundance statistics were used to count the total number of species, and an assessment of sample completeness was done for species richness through the R package iNEXT, which provides functions to compute and plot the seamless rarefaction and extrapolation sampling curves [65]. This latter analysis was performed for each main surface current system of the SPO, namely: Cape Horn current, East Australian Current, Humboldt Current System, South Equatorial Current, South Pacific Subtropical Gyre, and West Wind Drift, to evaluate whether some areas had a greater or lower coverage than others. The previously mentioned main surface current systems were downloaded from Harvard Dataverse [66].

We generated a biological-environmental spatial database, wherein each cell had the biogeographic information of copepod alpha and beta diversity together with oceanographic data from the selected environmental variables. We employed Generalized Additive Models (GAMs) to evaluate the relationship between copepod alpha and beta diversity in the 0–200 m layer of the South Pacific Ocean and the environmental predictors while considering spatial autocorrelation in our analyses. Spatial correlation is a pattern in which observations are related to one another by the geographic distance between the observation, with locations close to each other exhibiting more similar values than those further apart, thus violating the assumption of independent and identically distributed errors, which can inflate type I errors [67]. This can lead to the selection of unimportant explanatory variables and poorly estimated parameters, moreover in ecology, as autocorrelation is a general property of ecological variables measured over geographic space [67]. Therefore, to take spatial autocorrelation into account, we used the residuals autocovariate (RAC) approximation [67] by including autocorrelation in the GAMs by adding another term to it that represents the influence of neighboring observations on the dependent variable at a particular location. Subsequently, the residuals of the auto-covariables were used as a new predictive variable in the models. The calculations of GAM+RAC were performed with the spdep package [68, 69] implemented in R software (R Core Team, 2021).

The model selection was done through Bayesian Information Criterion (BIC), that is based on information theory but within a Bayesian context and imposes a greater penalty for the number of parameters compared to the Akaike information criterion [70]. The best model is the one that provides the minimum BIC, denoted by BIC*, and delta BIC can be computed as: ΔBIC = BIC_m_−BIC*. This implies that for given M models, the magnitude of the delta BIC can be interpreted as evidence against a candidate model being the best model [70]. As a rule, values of ΔBIC less than 2 are not worth more than a mention, values between 2 and 6 indicate that evidence against the candidate model is positive, values between 6 and 10 indicate that evidence against the candidate model is strong, and values greater than 10 indicate that evidence against the candidate model is very strong [70]. The analyses were conducted through the package MuMIn [71], in R software (R Core Team, 2021).

Beta diversity was evaluated at each side of the SPO using Generalized Dissimilarity Modelling (GDM)-based spatial analysis technique, which involves mapping the first three axes derived from a principal component analysis (PCA) performed on the set of transformed predictor layers [72]. It first formulates the relationship between biological dissimilarity and spatial distance by transforming each predictor variable through l-spline basis functions considering a term called ‘predicted ecological distance’ in a negative exponential function, with the assumption that dissimilarity between site-pairs increases monotonically from 0 to 1 and in a saturating manner with predicted ecological distance. Based on the maximum height of the spline function (and hence, the maximum value of the transformed predictor), which shows the importance of the predictor in explaining dissimilarities, only the predictors with maximum height over zero were selected for the generation and plotting of the spatial layers. A RGB (RedGreenBlue) color palette dimension was assigned to each of the three axes of the PCA, and they were mapped by combining them, finally enabling the visualization of areas predicted to have more similar species composition (shown as similar colors) or less similar species composition (shown as different colors). The GDM analysis was performed using the package gdm [73] in R software (R Core Team, 2021) and its plotting was done through Kriging interpolation using ArcMap 10.4.1 (ESRI 2016) software.

## Results

### Diversity patterns

For the South Pacific Ocean, a total of 178 species were reported at the upper 200 m water column of its basin. Regarding the main species present at each main surface current system, we searched the dominant ones in terms of their occurrence along the whole period for each of the major current systems defined above. Most of the dominant species in the SPO were representatives of the Order Calanoida (S1 Table), except for the cyclopoid *Oithona similis*, which was the dominant species in the CHC, along with *Calanus simillimus* and *Pleuromamma robusta*. In the EAC, *O*. *similis* and *C*. *simillimus* were the dominant species accompanied by *Neocalanus tonsus*. In the SPSG *Calocalanus kristalli*, *Paracalanus parvus*, and *Calocalanus plumulosus* dominated; in the WWD *Calocalanus kristalli*, *Paracalanus parvus* and *Calocalanus plumulosus* were the most recurrent species; in the HCS *Paracalanus indicus*, *Oithona similis* and *Acartia tonsa* prevailed; and finally in the SEC *Paracalanus parvus*, *Calocalanus kristalli* and *Calocalanus plumulosus* were the most dominant ones (S1 Table).

For beta diversity and its components turnover and nestedness, the list of dominant species is shown in S2 Table. The dominant species for the mean beta diversity were the Calanoids *Acartia longiremis*, *Calocalanus kristalli*, *Calocalanus pavo*, and *Paracalanus parvus*, whereas turnover had similar dominant species, except by the substitution of *C*. *pavo* by *C*. *plumulosus*. Finally, nestedness resulted in a different species assemblage represented by *Calocalanus pavo*, *Euterpina acutifrons*, *Lucicutia flavicornis*, and *Mecynocera clausi*.

**Fig 2:**
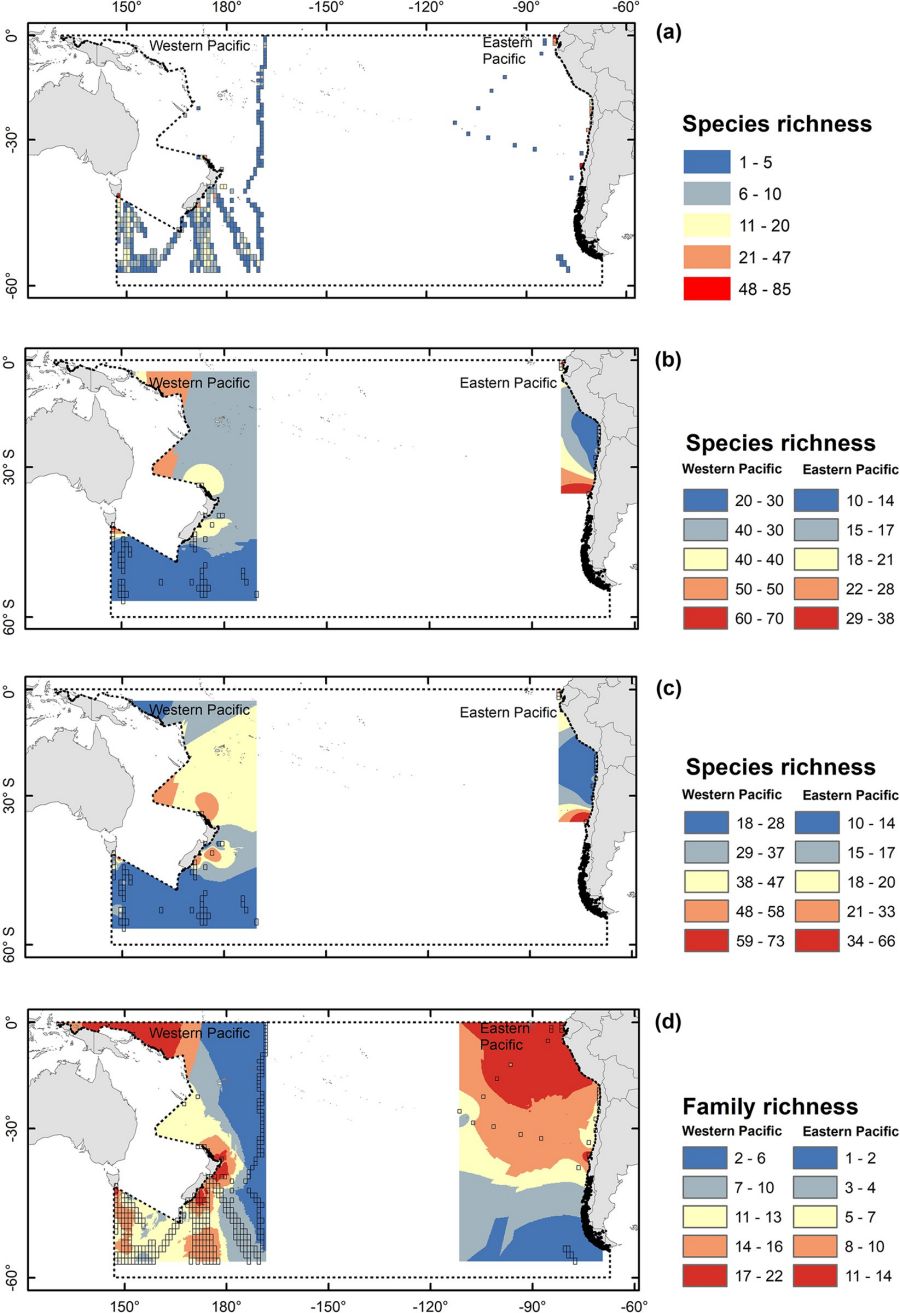
Richness patterns of Copepoda in the 0–200 m layer of the western and eastern side of the SPO for the period 1993–2019 based on species occurrence data obtained from OBIS portal. **(a)** Spatial distribution of alpha diversity considering the complete data cell with the curated data (i.e., 377 cells). **(b)** Inverse distance weighted (IDW) analysis for alpha diversity using cells with >10 species (i.e., only 77 cells from the total of 377 were selected). **(c)** Kriging interpolation for alpha diversity using cells with >10 species (i.e., only 77 cells from the total of 377 were selected). **(d)** Kriging interpolation for family richness. Transparent squares are the 1° sampled cells used for interpolation, whereas the grey dotted line delimits the South Pacific Ocean. Map projection is WGS 84/PDC Mercator (EPSG 3832).

The spatial distribution of alpha diversity considering the complete data set with the curated data (i.e., 377 cells, excluding the -110°−-170° longitudinal band) is shown in Fig 2A. The removal of cells with values of alpha diversity < 10, left 77 cells located at the eastern and western sides of the SPO, that were used for the inverse distance weighted (IDW) technique analysis (Fig 2B), and for the Kriging interpolation (Fig 2C). Those results showed a similar tendency as the ones obtained with Kriging interpolation done to explore the outcome of this approach by using the complete dataset (393 cells, without excluding the -110°−-170° longitudinal band), i.e., showing lower values of diversity at the western side of the SPO with a peak in the tropical and temperate zone, decreasing towards higher latitudes, whereas the eastern side showed higher values peaking at the temperate area that decreased towards the Equator and from the coastal towards the oceanic zone (Fig 2B and 2C). When considering family level, the number of cells increased to 399, and the Kriging interpolation presented a pattern with more families observed towards the eastern tropical and equatorial zone, with lower values towards the temperate, subpolar, and western area; whereas its western side showed higher values of families towards the continent with peaks in equatorial and temperate areas, and decreasing towards the open ocean (Fig 2D).When considering genus level, the number of cells increased to 394, and the Kriging interpolation presented a similar pattern to that observed at species level for eastern alpha diversity in Fig 2A and 2B, with clearer peaks in northern Chile and off Ecuador; whereas its western side showed higher values of genus towards the continent with peaks in equatorial and temperate areas, and decreasing towards the open ocean (S1 Fig).

**Fig 3:**
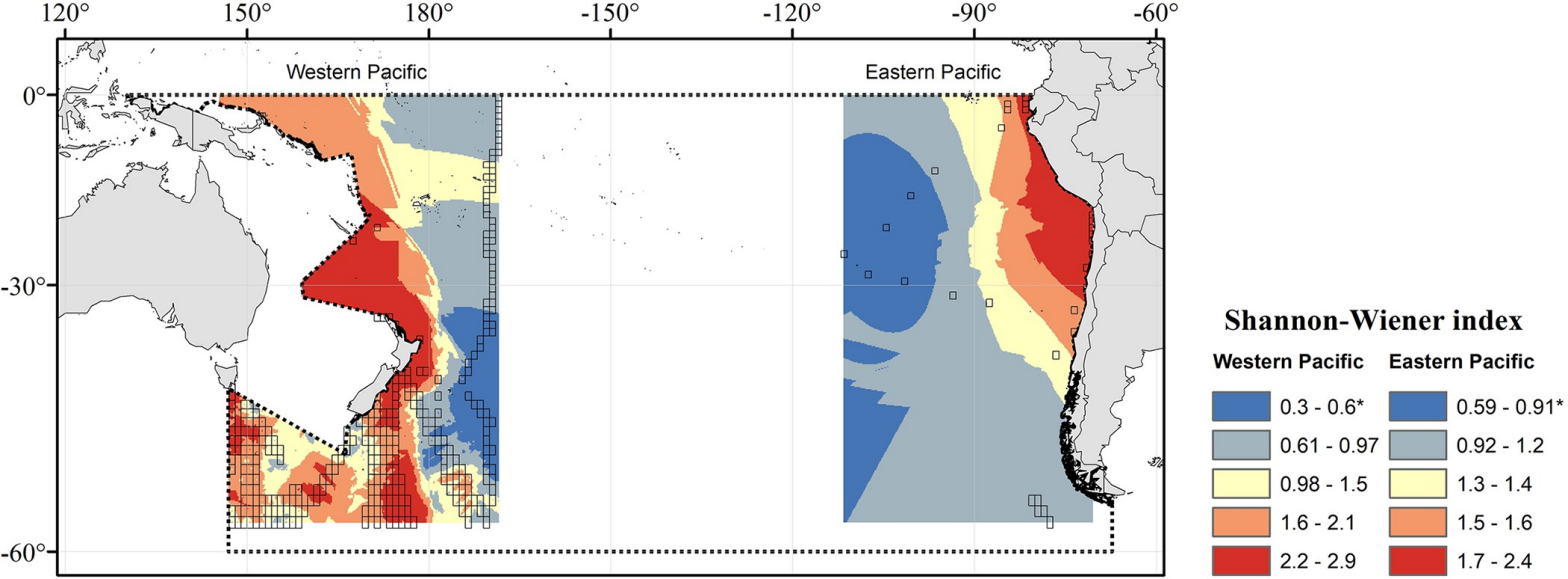
Shannon-Wiener index of Copepoda in the western and eastern sides of the South Pacific Ocean. Transparent squares are the 1° sampled cells used for Kriging interpolation, whereas the grey dotted line delimits the South Pacific Ocean. *Estimates based on available data at species level. Map projection is WGS 84/PDC Mercator (EPSG 3832).

The Shannon-Wiener index of diversity for pelagic copepods exhibited a pattern that varied from that of alpha diversity. For the eastern and western sides of the basin, the higher values were within the moderate range (2.4 and 2.9, respectively), showing different spatial patterns. At the eastern side of the SPO, greater diversity is observed on a coastal band from the temperate zone towards the equator, with decreasing values towards the open ocean (Fig 3). At the western side, higher diversity was observed from subtropical areas towards temperate and subpolar zones, while also showing decreasing values towards the open ocean (Fig 3).

**Fig 4:**
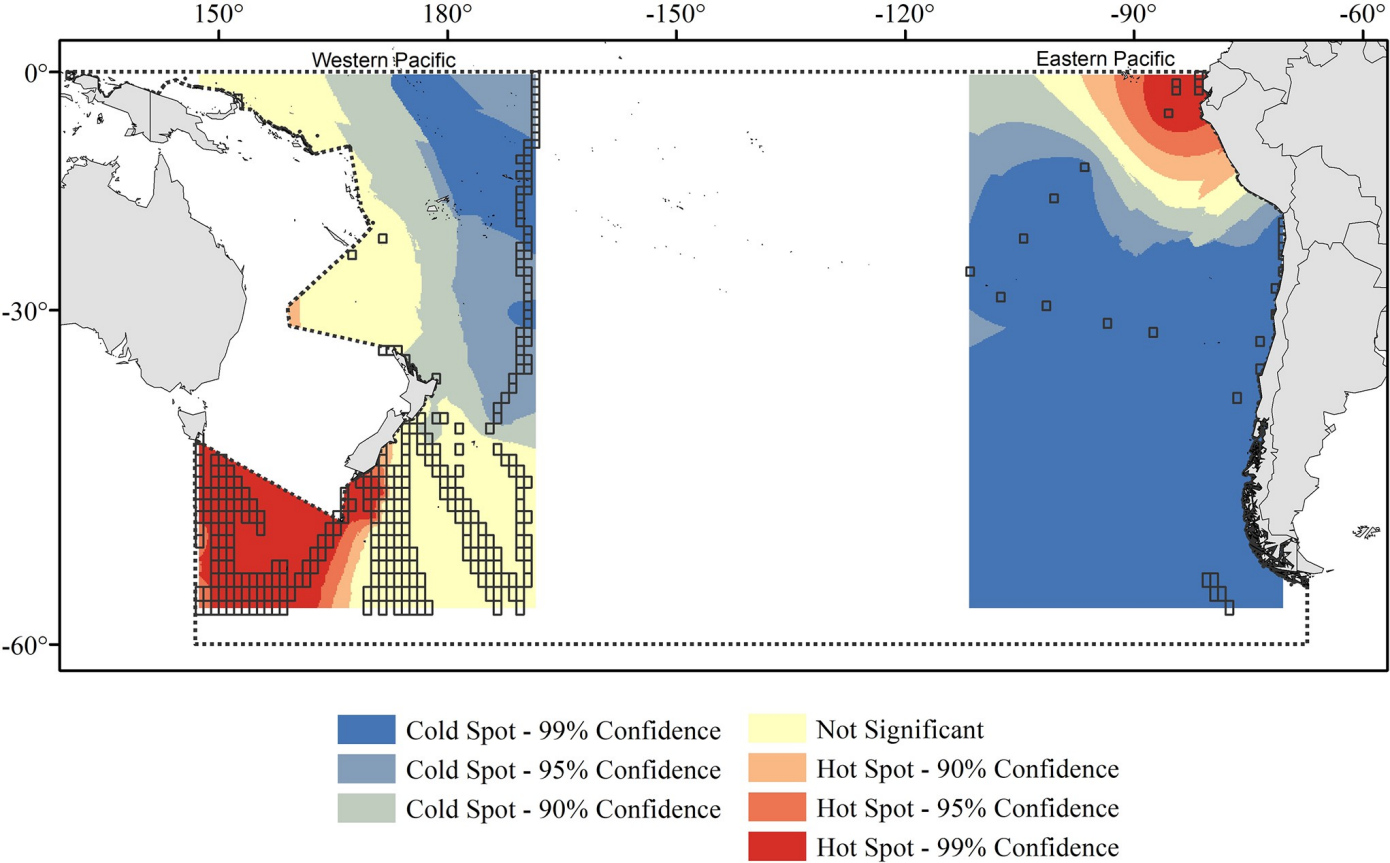
Getis-Ord G statistic of alpha diversity of Copepoda at the western and eastern sides of the SPO based on species occurrence data obtained from OBIS portal for the period 1993–2019. High and low values of richness are displayed in red and blue color, respectively. Color intensity denotes clusters’ significance. Transparent squares are the 1° sampled cells used for Kriging interpolation, whereas the grey dotted line delimits the South Pacific Ocean. Map projection is WGS 84/PDC Mercator (EPSG 3832).

To identify the extreme areas in terms of diversity, we used the High/Low Clustering (Getis-Ord G) statistical analysis. This method yielded patterns consistent with those of diversity (in terms or species richness) and allowed us to identify most of the hot spots (high values of richness) present in western temperate-subpolar areas off Australia, and in the equatorial area off Peru and Ecuador, decreasing towards the south and into the open ocean (Fig 4).

**Fig 5:**
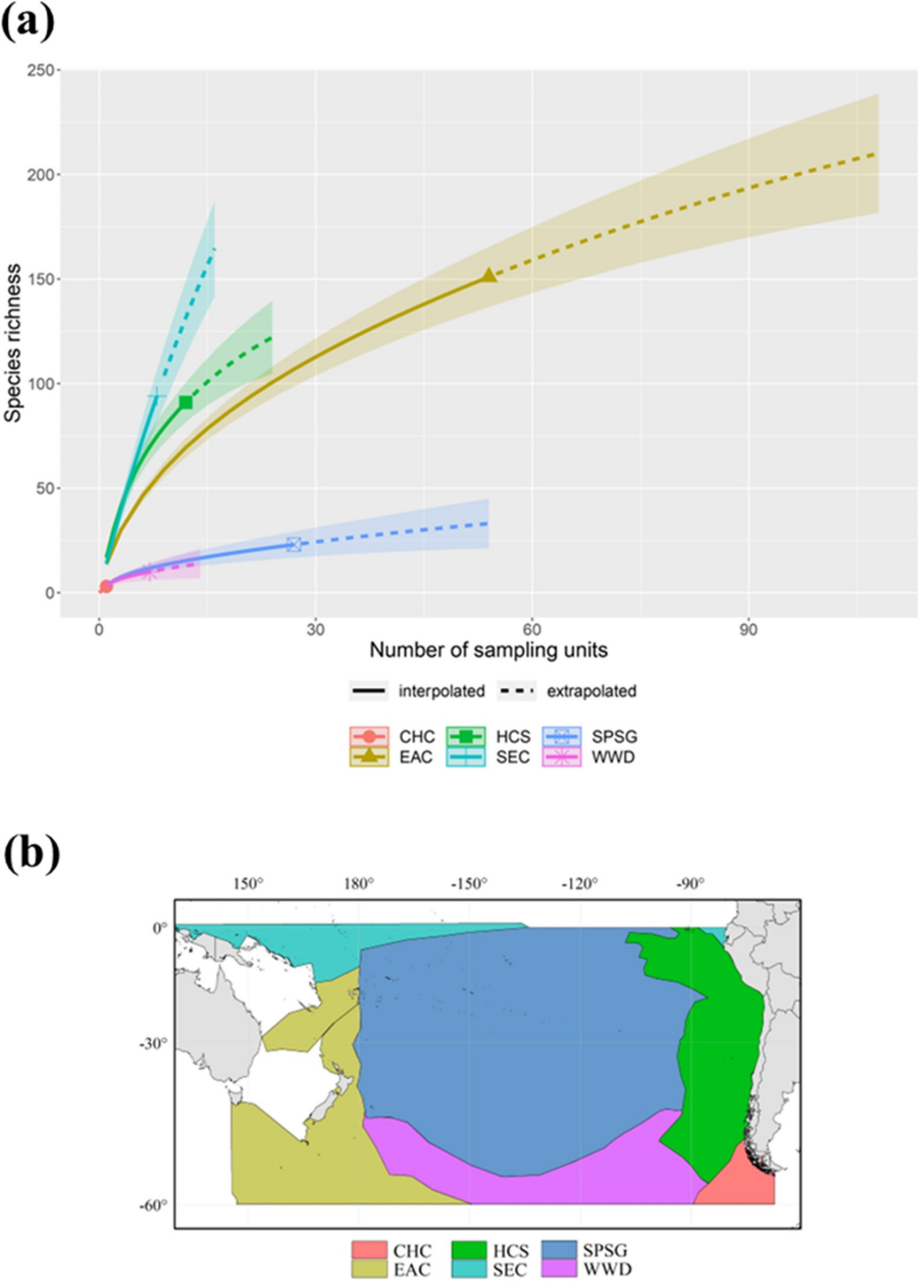
**(a)** Sample-size-based rarefaction (solid line segments) and extrapolation (dotted line segments) sampling curves for species richness with 95% confidence intervals (shaded areas) for Copepoda in the main surface current systems of the SPO. The symbols of each curve represent the reference samples. CHC = Cape Horn Current; EAC = East Australian Current; HCS = Humboldt Current System; SEC = South Equatorial Current; SPSG = South Pacific Subtropical Gyre; WWD = West Wind Drift. **(b)** Map of each main surface current system of the SPO.

The expected number of species, as a function of sampling units for different defined regions can illustrate the actual level of sampling coverage (Fig 5). As shown by species rarefaction curves, the sampling effort (samples per sampling units) varied extensively within and between regions, and no saturated curves were found in any of the regions, indicating a rather low coverage to assess the whole copepod community (Fig 5A). The distribution of the defined regions or current systems are illustrated in Fig 5B.

**Fig 6:**
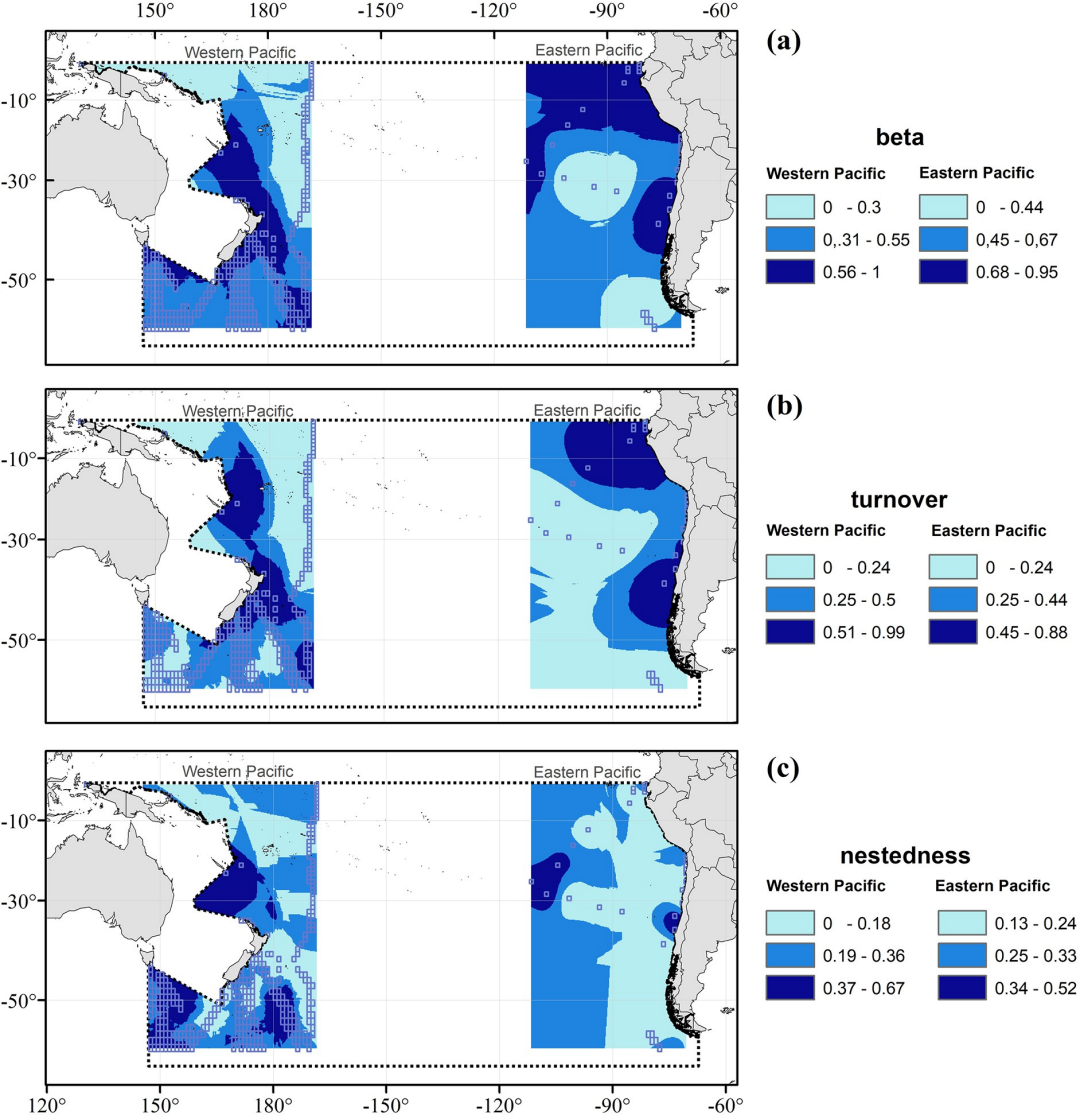
(a) Mean species composition and its components (b) turnover and (c) nestedness for Copepoda in the 0–200 m layer of the western and eastern sides of the SPO. Transparent squares are the 1° sampled cells used for Kriging interpolation, whereas the grey dotted line delimits the South Pacific Ocean. Map projection is WGS 84/PDC Mercator (EPSG 3832).

The spatial variability in diversity among potential distinct assemblies can be reflected in beta diversity, which may also indicate the degree to which such communities differ from each other. Beta diversity of copepods in the eastern SPO was higher in its equatorial-tropical area, with another area showing higher values at is temperate zone (Fig 6A); whereas the western side of the SPO had higher values of beta diversity expanding from tropical to subpolar areas (Fig 6A). This beta diversity (Fig 6A; mean beta = 0.579) was better explained by mean turnover (Fig 6B; mean turnover = 0.295) rather than mean nestedness (Fig 6C; mean nestedness = 0.284). The turnover rate at the eastern side of the SPO was higher in its equatorial-tropical area, with another area showing higher values at is temperate zone (Fig 6B); whereas the western side of the SPO had higher values of turnover expanding form tropical to subpolar areas (Fig 6B). Nestedness was found to be higher at the open ocean in the eastern side, with a higher values patch associated to the coastal area in the temperate zone (Fig 6C); whereas at the western side, it showed higher values in more oceanic areas at the temperate and subpolar zones (Fig 6C).

**Fig 7:**
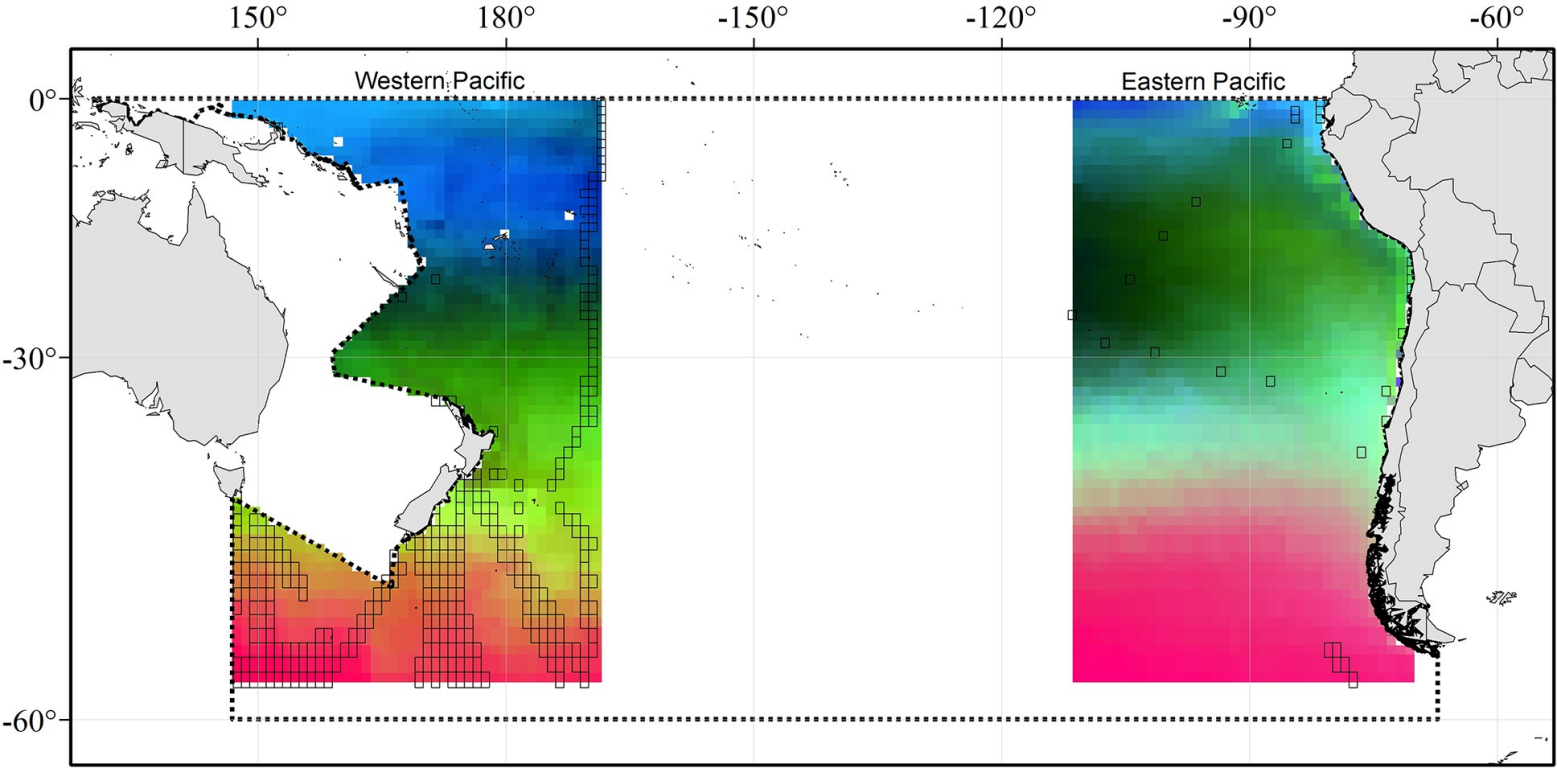
Predicted spatial variation in species composition of Copepoda at the western and eastern sides of the SPO based on a principal component analysis (PCA) of the Generalized Dissimilarity Modelling (GDM)-transformed environmental predictors. Colors represent gradients in species composition. Locations with similar colors indicate more similar expected composition. Dashed line at both sides of the antemeridian (180° longitude) delimits the South Pacific Ocean. Map projection is WGS 84/PDC Mercator (EPSG 3832).

When applying the GDM-based spatial analysis to assess species composition, we found that salinity was the strongest predictor of the observed dissimilarities, followed by oxygen concentration, mean temperature and chlorophyll-a concentration. In contrast, mixed layer depth, temperature stability and standard deviation of temperature were the least important predictors, (S2 Fig). The spatial layers were generated and plotted with the best predictors and showed three clear latitudinal bands of similar color that kept almost the same distribution when extracting the -110°−-170° longitudinal band, indicating a more similar expected composition of Copepoda or the existence of distinctive species assemblies (communities) with a variable degree of mix; however, they covered large areas over each side of the basin, indicating the existence of different communities (Fig 7). These distinct communities can also be defined in terms of their dominant species which are shown in S2 Table. Some of these dominant species are shared between current systems, although their presence also reflects a wide distribution over the SPO basin.

### Data coverage, biogeographic patterns, and dominant species

This assessment was carried out in association with the major current systems illustrated in Fig 1. The general circulation of the SPO is dominated by the subtropical gyre, that has an elevated mean dynamic topography at its center [74] (near 15° to 20°S [75]). The South Equatorial Current represents the westward flow of the South Pacific Subtropical Gyre, that terminates entering the Coral Sea and bifurcating at the east of the Great Barrier Reef between 15° and 22°S [76] to form the southerly East Australian Current [77], that strengthens as it flows along the coast of Australia [77]. Southward of ~33°S, it begins to separate into filaments, forming the East Australian Current extension and the Tasman Front [77] that consists of a series of current jets flowing eastward, mainly between 33° and 35°S [78]. These appears to be a large component of subtropical water feeding into the South Pacific Subtropical Gyre circulation along the Sub-Tropical Front associated with the West Wind Drift, that streams equatorward of the Subantarctic Front associated with the Antarctic Circumpolar Current (ACC) [79, 80] and intersects the South American continent at ~45°S, where the Cape Horn Current and Humboldt Current System begin towards the south and the north, respectively [81, 82]. The latter is the largest of the four main Eastern Boundary Upwelling Systems—as the other three are embedded in the California, Canary, and Benguela Currents [83]—and is shaped by a broad current of fresh, cooler Sub-Antarctic Surface Water along the eastern rim of the subtropical gyre [81], extending from southern Chile (~45°S) to northern Peru and Ecuador (~4°S), where cool upwelled waters collide with warm tropical waters forming the Equatorial Front [81]. The South Pacific Subtropical Anticyclone or the South Pacific High, which spins counter-clockwise, is the most influential off the west coast of South America [74, 84] and acts as the dominant forcing of the subtropical gyre [85]. However, it presents seasonal variation, abiding at its northern position (26°S, 86°W) during the late austral fall and winter, when it is also closer to the South American continent and its intensity is weaker; whereas, during austral spring and summer the South Pacific High moves southwest (37°S, 108°W) and depicts its maximum intensity [85]. The latter (former) conditions generate stronger (weaker) Equatorward winds that favor (disfavor) coastal upwelling offshore central-south Chile [81]. In terms of sampling coverage for epipelagic copepods, only 4.02% of the total area of the epipelagic SPO has been sampled between 1993 and 2019. In fact, all the main current systems have been sampled over less than 2.5% of their area, each covering less than 1% of the total area of the SPO. An exception is the East Australian Current System with 17.28% of its area sampled and covering 2.76% of the total area of the SPO (Table 1).

**Table 1:** Summary of spatial characteristics and cells sampled per each main surface current system of the SPO. CHC = Cape Horn Current; EAC = East Australian Current; HCS = Humboldt Current System; SEC = South Equatorial Current; SPSG = South Pacific Subtropical Gyre; WWD = West Wind Drift; n.a. = not applicable.

Current	Area (Km^2^)	Total number of cells	Number of sampled cells	Total number of observations	Number of species observed per current	Sampled area per current (Km^2^)	% of the current’s area sampled	% of the area of the SPO sampled
**CHC**	2.65 *10^6^	152	5	55	3	6.16 *10^4^	2.32	0.05
**EAC**	1.93 *10^7^	1468	270	18207	148	3.33 *10^6^	17.28	2.76
**HCS**	1.59 *10^7^	1230	16	2527	91	1.97 *10^5^	1.24	0.16
**SEC**	6.74 *10^6^	685	12	510	94	1.48 *10^5^	2.19	0.12
**SPSG**	6.14 *10^7^	4436	67	899	24	8.26 *10^5^	1.34	0.68
**WWD**	1.46 *10^7^	804	23	147	10	2.83 *10^5^	1.94	0.24
**Total**	1.21 *10^8^	8775	393	22345	n.a.	4.84 *10^6^	n.a.	4.02

#### Environmental data selection and predictive models

The routine VSURF selected five oceanographic factors to be tested for influence on species richness (arranged in decreasing order of importance): mean temperature (T_mean_), salinity (S), dissolved oxygen concentration (O_2_), chlorophyll-a concentration (Chla), and the mixed layer depth (MLD). For species composition, all seven oceanographic variables were selected (arranged in decreasing order of importance): mean temperature (T_mean_), chlorophyll-a concentration (Chla), dissolved oxygen concentration (O_2_), standard deviation of temperature (T_sd_), salinity (S), temperature stability (T_stab_), and mixed layer depth (MLD). The Spearman correlation matrices (S3A and S3B Fig) obtained for each subset of variables depicted that all were weakly correlated according to a Spearman’s rank correlation coefficient |ρ <0,7|, with exception of T_mean_ and O_2_, which showed ρ = -0.99.

However, in addition to environmental variables, data on Copepoda richness within the upper 200 m of the SPO depicted that the species and assemblies were significantly associated over the space according to Moran’s I statistics. This is shown by regression residuals which exhibited significant spatial autocorrelation, i.e., there are spatial-dependent elements in the regression residuals (I = 0.012, p-value = 0.015). Therefore, the residuals autocovariate (RAC) was added as a new variable for the model selection, after which the spatial autocorrelation according to Moran’s I statistics was not significant (I = 0.003, p = 0.091).

When looking for environmental predictors to explain the distribution of species richness, the best GAM explaining species richness included Chla, S, T_mean_ and RAC (Table 2). The adjusted R^2^ value revealed that 60% of the variability is explained by this model. The predicted values of alfa diversity were concordant with the observed data (r = 0.77; S4 Fig).

**Table 2:** GAM+RAC models for species richness. Statistics acronyms are BIC = Bayesian information criterion, ΔBIC = delta BIC (i.e., the difference in BIC score between the best model and the model being compared). Predictors’ acronyms are: T_mean_ = mean temperature, S = salinity, Chla = chlorophyll-a concentration, MLD = mixed layer depth, autocovariate = residuals autocovariate. The best model is highlighted in bold.

	Model	BIC	ΔBIC
**1**	**Species richness ~ Chla, S, T**_**mean**_ **+ autocovariate**	**2364.54**	**0**
2	Species richness ~ Chla, MLD, S, T_mean_ + autocovariate	2365.47	0.93

Regarding species composition within the 0–200 m range, a significant spatial correlation according to Moran’s I statistics was observed, as regression residuals exhibited significant spatial autocorrelation, (I = 0.018, p-value = 0.005). After we added RAC as a new variable for the model selection, spatial autocorrelation did not occur based on Moran’s I statistics (I = -0.006, p-value = 0.726).

The best GAM model explaining the species composition of copepods in the SPO included Chla, O_2_, S, T_mean_ and RAC (Table 3). The adjusted R^2^ value revealed that 70% of the variability is explained by this model. The predicted values of beta diversity were concordant with the observed data (r = 0.84; S5 Fig).

**Table 3:** GAM+RAC models for species composition. Statistics acronyms are BIC = Bayesian information criterion, ΔBIC = delta BIC (i.e., the difference in BIC score between the best model and the model being compared). Predictors’ acronyms are: T_mean_ = mean temperature, T_stab_ = temperature stability, S = salinity, Chla = chlorophyll-a concentration, DO_2_ = dissolved oxygen concentration, MLD = mixed layer depth, autocovariate = residuals autocovariate. The best model is highlighted in bold.

	Model	BIC	ΔBIC
**1**	**Species composition ~ Chla, DO**_**2**_**, S, T**_**mean**_ **+ autocovariate**	**553.32**	**0**
2	Species composition ~ Chla, MLD, DO_2_, S, T_mean_ + autocovariate	551.84	1.48
3	Species composition ~ Chla, MLD, DO_2_, S, T_mean_, T_stab_ + autocovariate	551.68	1.64

## Discussion

### Spatial patterns of biodiversity and their predictors

Hot and cold spots patterns of copepod distribution in the SPO do not seem to follow the previously reported latitudinal trends of global patterns of marine species with peaks of diversity at subtropical latitudes and a gradual decrease towards temperate and polar regions [23, 34, 86–90]. However, the presence of hot spots equatorial area off Peru and Ecuador (Fig 4) coincides with the Eastern margin biogeographic region where coastal (0–250 Km offshore) and eastern (250–1000 Km offshore) boundary currents that originate from the Humboldt Current and has high levels of production [91]; whereas the hotspots observed in western temperate-subpolar areas off Australia (Fig 4), corresponds, according to their zoning, to the South Subtropical Convergence and to the Chatham Rise zones, both frontal zones that marks the intersection of colder sub Antarctic waters with warmer tropical waters thus supporting substantial production [92]. The latitudinal trend, suggesting a plateau distribution of diversity from tropical to temperate regions, can be observed at the spatial distribution of the Shannon index shown in Fig 3, which shows greater diversity over a coastal band from the Equator towards the temperate area in the eastern zone, and higher diversity from subtropical areas towards temperate and subpolar zones at the western zone. Shannon Wiener, however exhibited some discrepancies with species richness. For instance, when comparing the northern vs. the southern portion of the eastern SPO (i.e. the Humboldt Current). In this regard, it is important to consider that we estimated the Shannon Wiener based on the species occurrences over the study period, not on species abundance, whereas species richness represents the total number of species throughout the study period, independently of their frequency of occurrence. Therefore, Shannon Wiener and species richness may show different patterns of diversity, when comparing regions where copepod populations are highly frequent, since their populations are more continuously present during their annual cycles, such as in tropical or subtropical regions, or they have very seasonal life cycles, with low frequency of occurrence, such as in temperate regions [93].

Regarding latitudinal trends, Woodd-Walker et al. (2002) [36] suggested that the decreasing trend with latitude may arise from a more stable seasonal cycle of productivity in the tropical/subtropical area supporting a stable high-diversity community in comparison with a strongly seasonal productivity in temperate/polar regions which may limit the number of copepods species and so resulting in a low diversity pattern. This latitudinal pattern is reflected in our spatial distribution of Shannon-Wiener index in both sides of the SPO, but it is not clear for the spatial distribution of alpha diversity, that at its western side showed lower values of diversity and had a similar tendency with a peak in the tropical and temperate zone, decreasing towards higher latitudes, although the eastern side showed higher values peaking at the temperate area that decreased towards the Equator and from the coastal towards the oceanic zone.

The zonal pattern on the other hand, remains with much uncertainty due to a poor sampling coverage in the oceanic region of the SPO. Our study shows that maximum values of copepod richness occur around tropical-subtropical areas in both eastern and western sides, but with a strongly reduced diversity in the oceanic region in areas with too few sampled cells. In this regard, Rombouts et al. (2009) [23] suggested that temperature-predicted diversity of copepods should be higher in a subtropical band in both hemispheres, although with no contrasting differences between coastal and oceanic regions. Such patterns have also been found in other planktonic groups, such as euphausiids [34], tintinnid ciliates [94], foraminifera [34, 95]; as well as benthic marine invertebrates, such as prosobranch gastropods [32]; and higher trophic level organisms, fishes [34, 96], sharks [34], squids [34] and cetaceans [34]. For instance, in a recent work [19] a total number of 121 species of copepods was reported from a single cruise carried out in the South Pacific Subtropical Gyre, indicating this area can be comparable to the North Pacific Subtropical Gyre in which 125 species were reported by McGowan and Walker (1979) [97], although more recently Vereshchaka et al. (2017) [98] indicated that more than 240 species can be found in the central-south Pacific region. Therefore, the species records available at OBIS may not represent the potentially rich copepod community in the central gyre of the SPO; and that is why we opted for dividing the basin into a eastern and western side and exclude the longitudinal band between -110°−-170° for performing our analyses, as this area had too few sampling.

Temperature and its stability were the main factors, initially proposed in our hypothesis, as predictors of copepod biodiversity patterns in this basin. However, hot spots were also found off Chile, Peru and Ecuador along the main coastal upwelling system, which might be associated to 1) the habitat features hypothesis [34] related to ecosystem size and mesoscale processes, that are known to influence positively copepod diversity [34, 86, 90]; and 2) the productivity-richness hypothesis that predicts a positive effect of primary productivity on richness [34], supported by other studies finding a positive relationship between copepod diversity and chlorophyll-a concentration [86, 87, 89, 99]. Biogeographic patterns can also be explained on the basis of evolutionary [100, 101] and spatial processes [102] which are considered as the historical context for observed spatial distribution. Nevertheless, as stressed above, limited data coverage precludes a clear conclusion on underlying mechanisms explaining observed patterns of diversity.

Considered the above-mentioned potential mechanisms, we selected environmental variables to evaluate factors explaining copepod species richness and species composition. All variables were correlated to each other with a Spearman’s rank correlation coefficient of |ρ <0,7|, except for T_mean_ and O_2_, that showed ρ = -0.99. In general, variables with correlation values of |ρ| less than 0.7 are not recommended for selection [103]; nevertheless, despite this high value of |ρ|, we chose to include both variables instead of conducting further testing. This is because dissolved gases in the ocean tend to decrease their concentration with higher temperature [104], and so this relationship would not reflect a systematic bias. The oceanographic factors explaining copepod species richness (Chla, S, T_mean_) and species composition (Chla, O_2_, S, T_mean_) in the SPO are of significance in a lesser or greater degree at different zones of the study area; and, they have also been documented to be of importance on a macroscale basis for copepod abundance and body size [25]. Moreover, the GDM-based spatial analysis conducted for evaluating species composition showed that the stronger predictors of the observed dissimilarities were the same variables (S, O_2_, T_mean_, Chla; S3 Fig).

It has been found that chlorophyll strongly correlates with copepod abundance in the ocean [34, 86, 99, 105]. Oxygen is also a significant factor influencing copepod distribution in the ocean, because its availability is a major driver for temperature-size responses in aquatic organisms [106], and the variation in its concentration, for example, due to deoxygenation, can affect copepod physiology, with drastic impacts in both coastal [11, 107] and oceanic [108] species. Warmer water increases copepod growth, development and molting rates [107], with an associated increase in the oxygen demand. This can lead to a reduction in population growth by limiting the hatching success and naupliar growth in early life stages [11], as well as reducing egg production and somatic growth in adults [107], together with shifts in their depth distribution [108]. This involves physiological adaptations that may vary according to the species, their body weight and extent of motion [109]. Altogether, these combined drivers have a potential effect on copepod ecology and, consequently, on their biogeographic patterns.

Salinity and its variation also affects copepod distribution [110], biomass [111], reproduction [112, 113], growth [114, 115], body size [25] or fatty acid synthesis [115], with various ranges of tolerance that may or may not be favorable, according to their biology and location. This may be relevant since global warming in some areas of the ocean can lead to an increase in salinity (evaporation or greater circulation) or a reduction of the same (greater run-off of fresh water into the ocean, rainfall, or ice melt), producing osmotic stress that may be costly for their physiology.

Temperature itself plays a fundamental role in the marine ecosystem, since it controls physiological rates [116], metabolism [116], body size [25], reproduction [10], mortality [117] and community structure [24, 118], even at large spatial scales [25, 35, 41, 119]. Thus, it may be thought that temperature also influences the distribution of organisms in the ocean, as it can substantially vary in its three dimensions. Over the meridional axis, temperature exhibits a strong gradient from the equator to the poles, with a similar gradient commonly present from coastal areas to central gyres, whereas in the vertical axis, it has a strong temperature gradient from the upper mixed layer down to the deep, cold waters. These gradients in the water column structure may contribute to the generation of large-scale spatial trends in the distribution of organisms such as copepods [120]. Although this study did not separate copepods into functional groups according certain traits more specifically linked to environmental variations, the fact that composition changed mainly due to oceanographic factors that influence the nesting of functional groups, highlights and support those findings [121], as beta diversity is a proxy of difference in number of species among ecosystems, thus reflecting changes or gradients in the environment as well.

Species distribution and the structure of species assemblages may additionally obey some physical processes (e.g., large-scale circulation, currents, eddies, fronts, interaction with the atmosphere) and the effect of local conditions. In this sense, it is possible that in addition to more direct effects on copepod populations, temperature is reflecting variability in water masses distribution controlled by large-scale circulation. The distribution of drifting plankton, including copepods, is largely affected by near-surface currents, allowing species radiation and colonization processes [122], and so influencing diversity patterns.

Regarding the structure of the copepod community, we found that some dominant species were representing different current systems, and thus distinct plankton communities. However, we could only assess most occurrences of species (not abundance) for each main surface current system of the SPO (S1 Table). The occurrences distribution of dominant species seemed to be concordant with patterns documented for copepod diversity on a large scale, with cyclopoids showing greater diversity in the subtropics and calanoids in the temperate zone [25, 123]. Dominant species in the each of the major currents are mostly represented by small Calanoid copepods and the widely distributed Cyclopoid *Oithona similis*. Some of the recurrent species are *Paracalanus parvus* and *P*. *indicus*. Both species are possibly a single species traditionally known as *P*. *parvus*, but morphologically close to *P*. *indicus*, although most likely being a new, non-described species [124]. Lately, it has been referred as *P*. *cf*. *indicus* in the Eastern SPO [125]. The other recurrent species are *Calocalanus* spp. They are also known as small-sized copepods and mostly found in oceanic waters [126]. The high occurrence of small-sized oceanic copepods, and low presence of large-sized Calanoids, such as *Calanus* spp., *Calanoides* spp., and *Pleurommama* spp., which are abundant in coastal areas of the SPO [38, 93] may indicate that small copepods prevail, or are numerically more abundant than large-sized ones. Also, most data sets do not include harpacticoids or other cyclopoid copepods, such as *Oncaea* spp. which may be highly abundant in oligotrophic waters of the SPO [127]. The OBIS data base does not include the observed number of species in each of the surface current systems (see S1 Table) when compared to total species inventories. For example, the maximum species richness available in OBIS for the East Australian Current (EAC) and the Humboldt Current System (HCS) is 148 and 91, respectively (see Table 1); that account for ca. a quarter of the species inventory compiled by the Banyuls Observatory data base on copepods for those areas [128]. In the other current systems, similar levels of differences between OBIS data and species inventories can be applied.

### Current data limitations over space and time

Sampling biases must be considered when validating the robustness of the derived spatial patterns and the role of environmental drivers. For example, it is crucial to consider that due to spatial gaps of data (Fig 1, Table 1), our results may be strongly constrained be sampling coverage [129, 130].

The spatial sampling effort was evaluated over the SPO through rarefaction/extrapolation curves by grouping the cells into the main surface current systems of the SPO (Fig 5A). It was found that species richness counts did not reach a clear asymptote at any of the defined current systems. Thus, the SPO is under-sampled for copepods. This aspect may have had an influence on Shannon-Wiener index values (Fig 3), as moderate to high values (from 1.7 on) were estimated only for a small area of the SPO. Subsequently, large areas exhibiting much lower values of richness and biodiversity may result from lack of sampling, description, and digitization of copepod data rather than having a relatively low diversity of species on a significant part of its extension. Moreover, the GDM-based spatial analysis done for evaluating species composition was performed with ecological data based on occurrence instead of abundance, which requires the assumption that any location where the species has not been observed can be treated as absence; therefore, making under-sampled locations more problematic [72]. Although invertebrates are the most common group documented after plants [131], and that an increasing number of data papers over time reflects progress in digitization and online platforms development for reporting observations that serve for biodiversity research [131, 132], it has been estimated that less than 10% of specimens’ registers are digitized [133], with even less data available online [134]. The lack of description and cataloguing of species and the incomplete knowledge regarding their geographic distribution (i.e., Linnean and Wallacean shortfalls, respectively) are among the most common setbacks for biodiversity research using databases [130], representing a practical limit for biodiversity knowledge that may lead to misidentification of ecological processes [130]. We further addressed this issue by estimating redundancy index, that indicated a middling sampling over the SPO (S6 Fig) with a lower coverage on its eastern side, that highlights the gaps on great extensions and depicts higher values for areas where a greater sampling effort was seen. Moreover, through Moran’s I index, it is possible to infer that the distribution of richness is more spatially clustered than expected if underlying spatial processes were random, as biodiversity aspects of species and communities are linked to complex interactions between physical, chemical, and biological factors. Considering this, and the developments in statistical modeling making possible to account for biases within heterogeneous data [135], we chose to use the RAC approximation [67] for the GAM analyses. This allows the incorporation of another variable into the models to take spatial autocorrelation into account and maintain strong predictive and inferential performance [67]. However, despite the usefulness of these methodologies, more effort is needed for improving the number and quality of distribution records used as primary data in macroecological studies [136], with databasing focused on georeferencing the information obtained from data collections [129].

### Copepod resilience to climate forcing

Copepod diversity may have remained stable over a long time and large space due to a strong resilience to environmental variability. In this aspect and when dissecting species composition into turnover and nestedness, we observed that the former was higher in areas where hot spots were also identified (Figs 6 and 4, respectively), coinciding with the flow of cold currents (Humboldt current, Zeehan Current in southern Australia, and southern part of the East Australian current off New Zealand into the West Wind Drift). Therefore, basin-scale ocean currents seem to be an important oceanographic driver explaining the distribution of copepods in the SPO. The potential of large-scale ocean currents generating distributional patterns at various levels in the ocean has recently been studied more across different taxa [137–141], showing results that support the hypothesis and suggest that it may be a general process transcending taxa and spatial scales [141]. All these large-scale circulation patterns may allow the recolonization and mixing of copepod assemblies, thereby acting as a buffer to cope with the local impact of warming, deoxygenation, or other altered environmental conditions driven by natural perturbations or anthropogenic origin. This buffering effect may allow planktonic organisms such as copepods to exhibit a high resilience to large-scale changes in hydrographic conditions due to global warming.

## Conclusion

The diversity distribution of copepods in the SPO follows general patterns observed in other organisms in the ocean, although patterns over a basin scale with presence of hot and cold spots do not replicate observed patterns reported for the North Pacific or Atlantic Ocean. The key environmental correlates for such biodiversity patterns were found to be mean temperature, chlorophyll-a concentration, salinity, oxygen concentration, and residuals autocovariate. A significant role for causing and maintaining copepod diversity patterns was also attributed to large-scale circulation processes, which may act as a buffer for changes in local conditions and allow species recolonization. Therefore, we suggest that planktonic copepods may exhibit a strong resilience to climate change impact.

However, caution should be taken in interpreting our findings since our modeling approaches and spatial analyses were strongly constrained by spatial and temporal gaps in sampling efforts and data availability. Future analyses are expected to improve observed patterns with the completion of global biodiversity data bases.

## Supporting information

S1 Fig Kriging interpolation analysis done for the western and eastern side of the SPO with 394 cells accounting for number of genera. Transparent squares are the 1° sampled cells used for Kriging interpolation, whereas the grey dotted line delimits the South Pacific Ocean. Map projection is WGS 84/PDC Mercator (EPSG 3832).
(JPG)

S2 Fig Maximum height of the spline function (hence the maximum value of the transformed predictors), indicating the stronger predictors of the observed dissimilarities for the Generalized Dissimilarity Modelling (GDM)-based spatial analysis. Their acronyms are: s = salinity, o2 = dissolved oxygen concentration, tmean = mean temperature, chla = chlorophyll-a concentration, mld = mixed layer depth, tstab = temperature stability, tsd = standard deviation of temperature. The spatial layers were generated and plotted with the predictors with heights over zero (i.e., s, o2, tmean and chla).
(JPG)

S3 Fig Spearman correlation matrices of environmental variables used in GAM models for (a) alfa diversity and (b) beta diversity. Positive correlations are displayed in red and negative correlations in blue color. Color intensity is proportional to the correlation coefficients. In the right side of the correlogram, the legend color shows the correlation coefficients and the corresponding colors. Their acronyms are: Tmean = mean temperature, Tstab = temperature stability, Tsd = standard deviation of temperature, S = salinity, Chla = chlorophyll-a concentration, O2 = dissolved oxygen concentration, MLD = mixed layer depth.
(JPG)

S4 Fig Correlation between observed and predicted species richness. Pearson’s r coefficient: 0.77 (p-value<0.01). Negative residuals (below the reference line) indicate knowledge shortfalls, whereas positive residuals (above the reference line) indicate underestimated species richness.
(JPG)

S5 Fig Correlation between observed and predicted species composition. Pearson’s r coefficient: 0.84 (p-value<0.01). Negative residuals (below the reference line) indicate knowledge shortfalls, whereas positive residuals (above the reference line) indicate underestimated species composition.
(JPG)

S6 Fig Redundancy index obtained for the western and eastern side of the SPO. Values close to 1 indicate good sampling, whereas values close to 0 indicate poor sampling. Transparent squares are the 1° sampled cells used for Kriging interpolation, whereas the grey dotted line delimits the South Pacific Ocean. Map projection is WGS 84/PDC Mercator (EPSG 3832).
(JPG)

S1 Table Dominant copepod species in terms of occurrence for the main surface current systems of the South Pacific Ocean. CHC = Cape Horn Current; EAC = East Australian Current; HCS = Humboldt Current System; SEC = South Equatorial Current; SPSG = South Pacific Subtropical Gyre; WWD = West Wind Drift.
(DOCX)

S2 Table Dominant copepod species of the South Pacific Ocean in terms of occurrence for all ranges values of beta diversity and its components turnover and nestedness. (DOCX)
