# Supplementary figures and images for: Biodiversity patterns of epipelagic copepods in the South Pacific Ocean: Strengths and limitations of current data bases
Source: PLoS One. 2024 Jul 11;19(7):e0306440. doi: 10.1371/journal.pone.0306440 (PMC11238982; doi:10.1371/journal.pone.0306440)

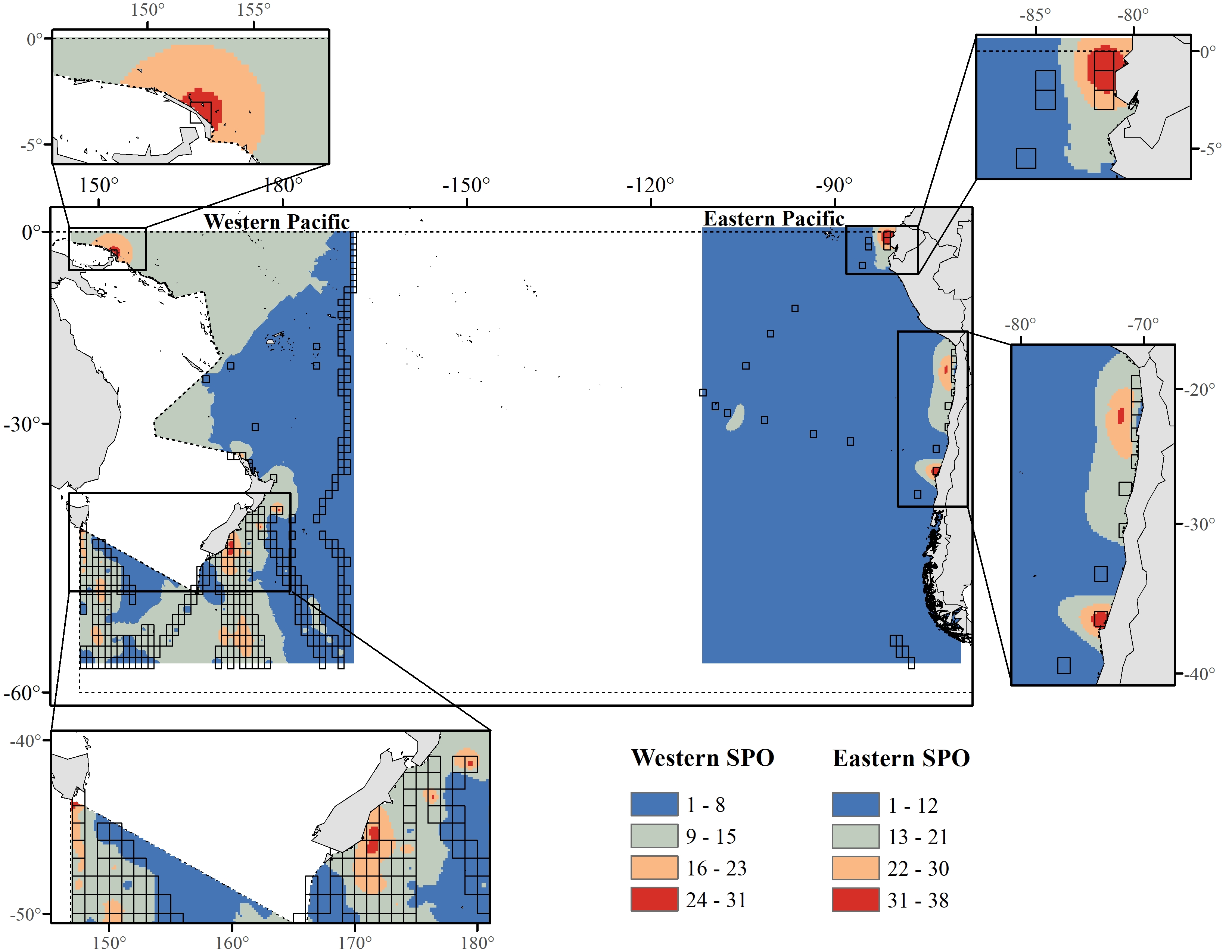

Supplement: S1 Fig — Transparent squares are the 1° sampled cells used for Kriging interpolation, whereas the grey dotted line delimits the South Pacific Ocean. Map projection is WGS 84/PDC Mercator (EPSG 3832). (JPG) [file pone.0306440.s001.jpg]

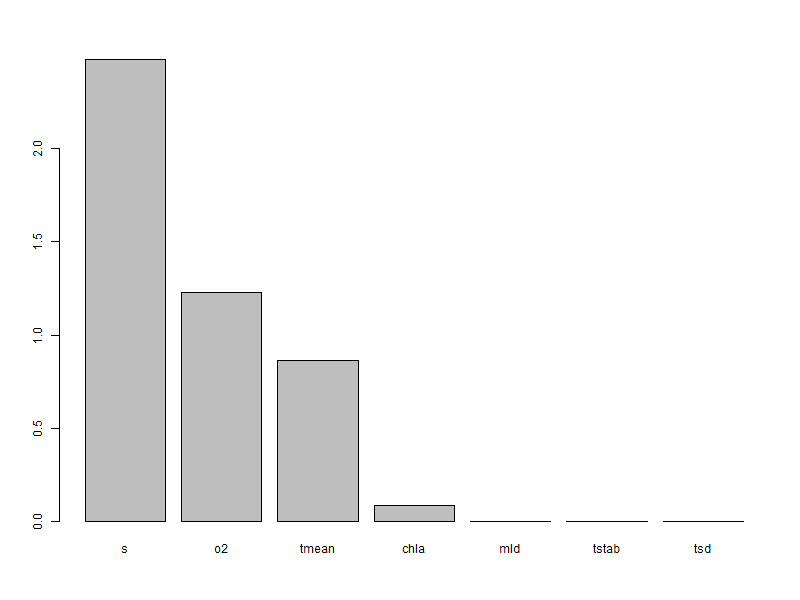

Supplement: S2 Fig — Their acronyms are: s = salinity, o2 = dissolved oxygen concentration, tmean = mean temperature, chla = chlorophyll-a concentration, mld = mixed layer depth, tstab = temperature stability, tsd = standard deviation of temperature. The spatial layers were generated and plotted with the predictors with heights over zero (i.e., s, o2, tmean and chla). (JPG) [file pone.0306440.s002.jpg]

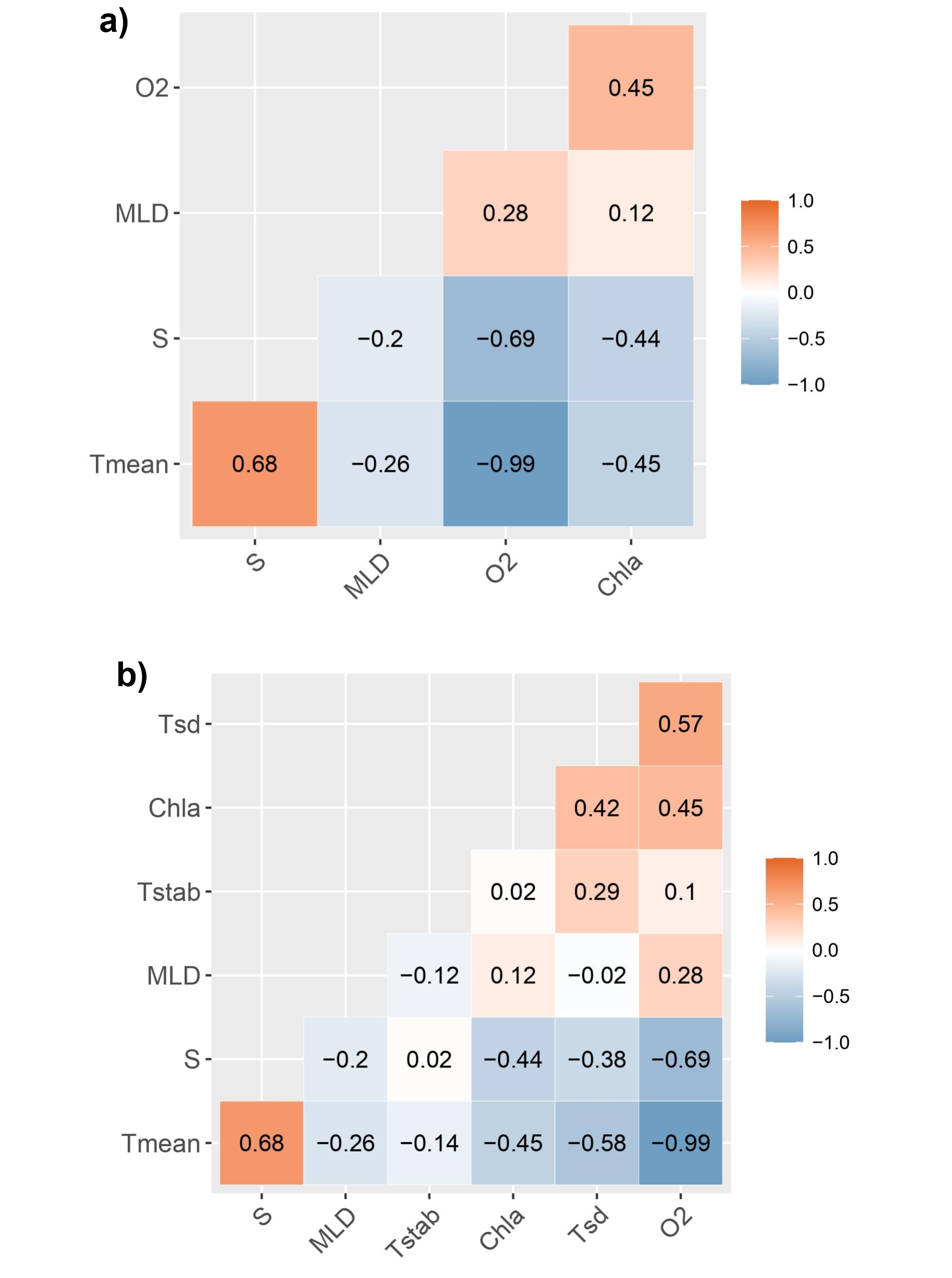

Supplement: S3 Fig — Spearman correlation matrices of environmental variables used in GAM models for (a) alfa diversity and (b) beta diversity. Positive correlations are displayed in red and negative correlations in blue color. Color intensity is proportional to the correlation coefficients. In the right side of the correlogram, the legend color shows the correlation coefficients and the corresponding colors. Their acronyms are: Tmean = mean temperature, Tstab = temperature stability, Tsd = standard deviation of temperature, S = salinity, Chla = chlorophyll-a concentration, O2 = dissolved oxygen concentration, MLD = mixed layer depth. (JPG) [file pone.0306440.s003.jpg]

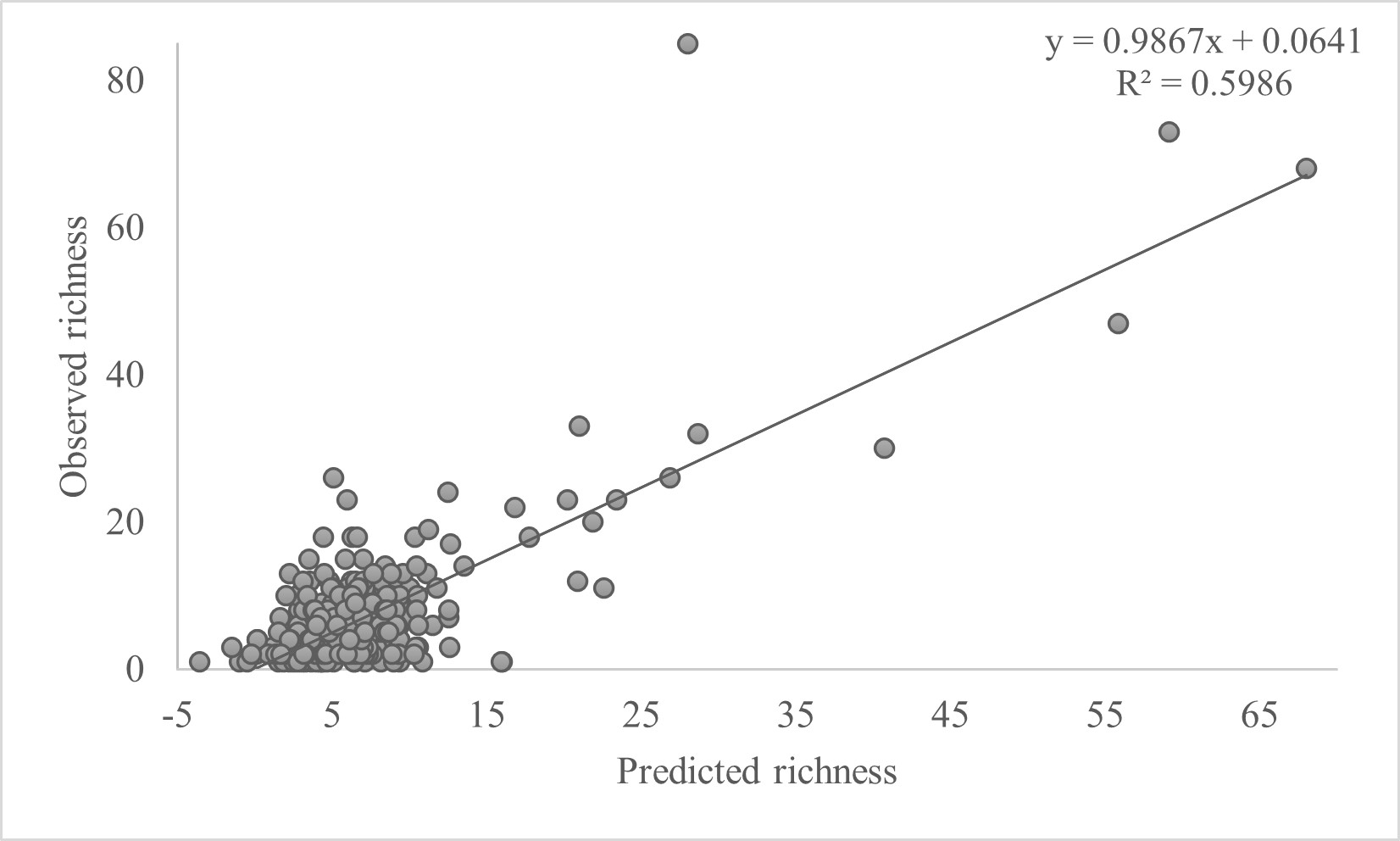

Supplement: S4 Fig — Pearson’s r coefficient: 0.77 (p-value<0.01). Negative residuals (below the reference line) indicate knowledge shortfalls, whereas positive residuals (above the reference line) indicate underestimated species richness. (JPG) [file pone.0306440.s004.jpg]

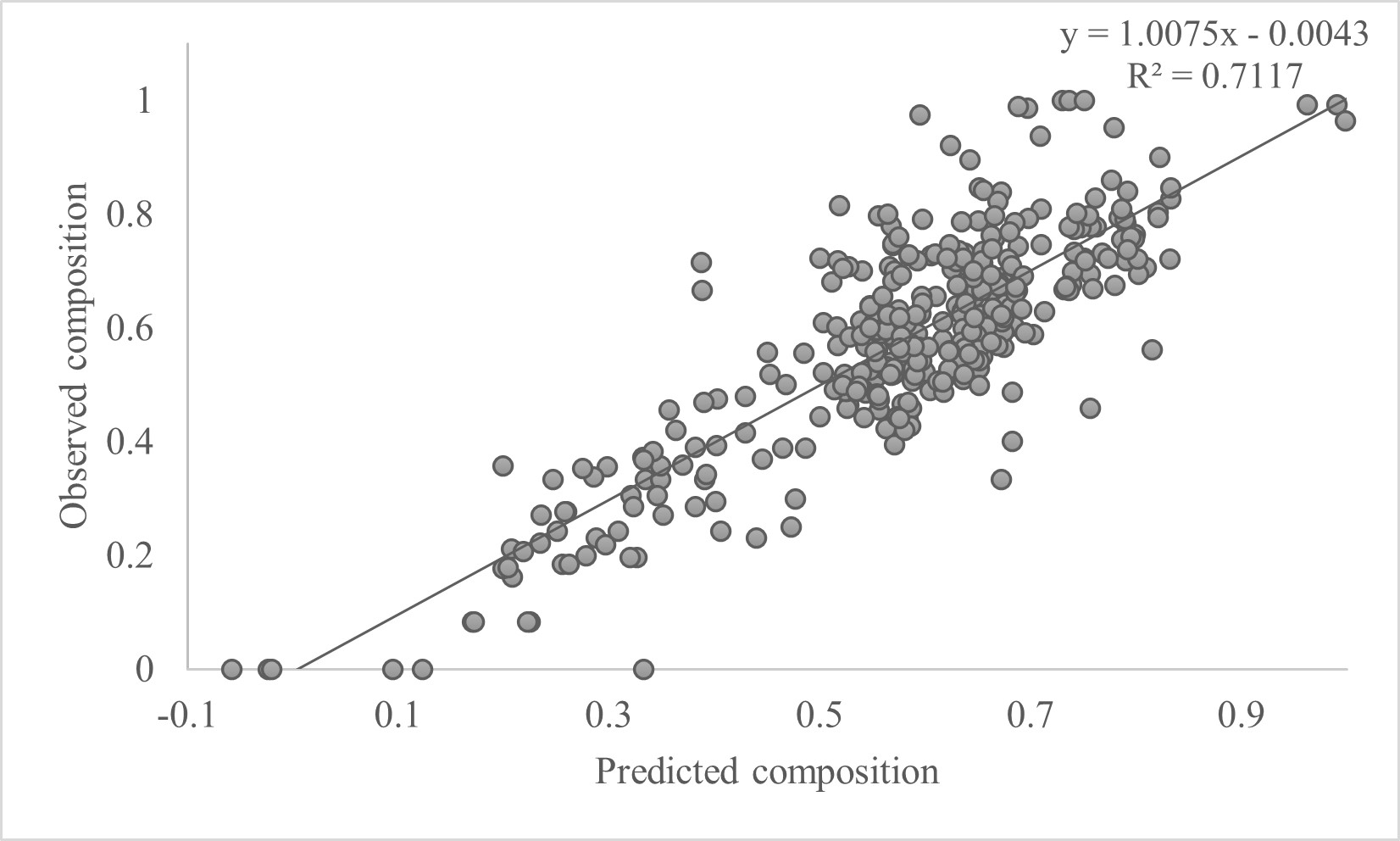

Supplement: S5 Fig — Pearson’s r coefficient: 0.84 (p-value<0.01). Negative residuals (below the reference line) indicate knowledge shortfalls, whereas positive residuals (above the reference line) indicate underestimated species composition. (JPG) [file pone.0306440.s005.jpg]

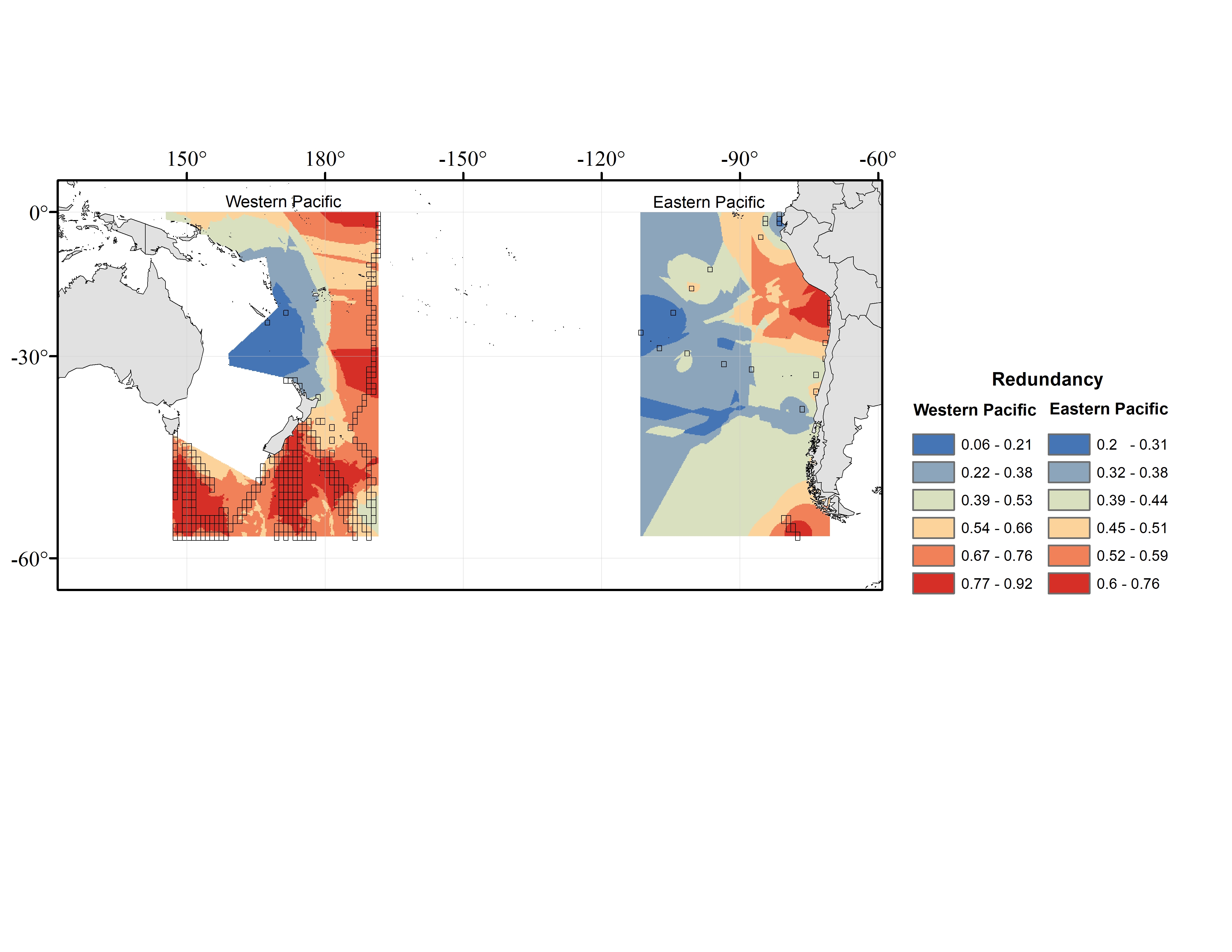

Supplement: S6 Fig — Values close to 1 indicate good sampling, whereas values close to 0 indicate poor sampling. Transparent squares are the 1° sampled cells used for Kriging interpolation, whereas the grey dotted line delimits the South Pacific Ocean. Map projection is WGS 84/PDC Mercator (EPSG 3832). (JPG) [file pone.0306440.s006.jpg]
