# Supplementary material for: Biodiversity patterns of epipelagic copepods in the South Pacific Ocean: Strengths and limitations of current data bases
Source: PLoS One. 2024 Jul 11;19(7):e0306440. doi: 10.1371/journal.pone.0306440 (PMC11238982; doi:10.1371/journal.pone.0306440)
Supplement: S1 Table — CHC = Cape Horn Current; EAC = East Australian Current; HCS = Humboldt Current System; SEC = South Equatorial Current; SPSG = South Pacific Subtropical Gyre; WWD = West Wind Drift. (DOCX) [file pone.0306440.s007.docx]

|  | Species name | Order | % of occurrence |
| --- | --- | --- | --- |
| CHC | *Oithona similis*  *Calanus simillimus*  *Pleuromamma robusta* | Cyclopoida  Calanoida  Calanoida | 61.8  36.4  1.8 |
| EAC | *Oithona similis*  *Calanus simillimus*  *Neocalanus tonsus* | Cyclopoida  Calanoida Calanoida | 26.9  11.0  8.9 |
| HCS | *Paracalanus indicus*  *Oithona similis*  *Acartia tonsa* | Calanoida  Cyclopoida  Calanoida | 6.5  5.9  5.8 |
| SEC | *Paracalanus parvus*  *Calocalanus kristalli*  *Calocalanus plumulosus* | Calanoida  Calanoida  Calanoida | 18.4  11.1  11.1 |
| SPSG | *Calocalanus kristalli*  *Paracalanus parvus*  *Calocalanus plumulosus* | Calanoida  Calanoida  Calanoida | 42.2  29.1  14.6 |
| WWD | *Calocalanus kristalli*  *Paracalanus parvus*  *Calocalanus plumulosus* | Calanoida  Calanoida  Calanoida | 59.9  27.9  4.1 |
