# Supplementary material for: Biodiversity patterns of epipelagic copepods in the South Pacific Ocean: Strengths and limitations of current data bases
Source: PLoS One. 2024 Jul 11;19(7):e0306440. doi: 10.1371/journal.pone.0306440 (PMC11238982; doi:10.1371/journal.pone.0306440)
Supplement: S2 Table — (DOCX) [file pone.0306440.s008.docx]

|  | Species name | Order |
| --- | --- | --- |
| Beta | *Acartia longiremis*  *Calocalanus kristalli*  *Calocalanus pavo*  *Paracalanus parvus* | Calanoida  Calanoida  Calanoida  Calanoida |
| Turnover | *Acartia longiremis*  *Calocalanus kristalli*  *Calocalanus plumulosus*  *Paracalanus parvus* | Calanoida  Calanoida  Calanoida  Calanoida |
| Nestedness | *Calocalanus pavo*  *Euterpina acutifrons*  *Lucicutia flavicornis*  *Mecynocera clausi* | Calanoida  Harpacticoida  Calanoida  Calanoida |
|  |  |  |
